# The Role of Complex Analysis in Modelling Economic Growth †

**DOI:** 10.3390/e20110883

**Published:** 2018-11-16

**Authors:** Angelica Sbardella, Emanuele Pugliese, Andrea Zaccaria, Pasquale Scaramozzino

**Affiliations:** 1 Department of Economics and Finance, Università degli Studi di Roma Tor Vergata, 00133 Rome, Italy; 2 ISC-CNR—Institute of Complex Systems, 00185 Rome, Italy; 3 School of Finance and Management, SOAS University of London, London WC1H 0XG, UK; 4 European Commission, Joint Research Centre (JRC), Seville, Spain

**Keywords:** economic fitness, complexity, capabilities, economic growth

## Abstract

Development and growth are complex and tumultuous processes. Modern economic growth theories identify some key determinants of economic growth. However, the relative importance of the determinants remains unknown, and additional variables may help clarify the directions and dimensions of the interactions. The novel stream of literature on economic complexity goes beyond aggregate measures of productive inputs and considers instead a more granular and structural view of the productive possibilities of countries, i.e., their capabilities. Different endowments of capabilities are crucial ingredients in explaining differences in economic performances. In this paper we employ economic fitness, a measure of productive capabilities obtained through complex network techniques. Focusing on the combined roles of fitness and some more traditional drivers of growth—GDP per capita, capital intensity, employment ratio, life expectancy, human capital and total factor productivity—we build a bridge between economic growth theories and the economic complexity literature. Our findings show that fitness plays a crucial role in fostering economic growth and, when it is included in the analysis, can be either complementary to traditional drivers of growth or can completely overshadow them. Notably, for the most complex countries, which have the most diversified export baskets and the largest endowments of capabilities, fitness is complementary to the chosen growth determinants in enhancing economic growth. The empirical findings are in agreement with neoclassical and endogenous growth theories. By contrast, for countries with intermediate and low capability levels, fitness emerges as the key growth driver. This suggests that economic models should account for capabilities; in fact, describing the technological possibilities of countries solely in terms of their production functions may lead to a misinterpretation of the roles of factors.

## 1. Introduction

Why are some countries wealthier than others, and why do some countries exhibit sustained rates of growth over long periods, whereas others appear to be stuck in a low-income, low-growth path? These questions have been central to economics ever since its origin as a science, following Adam Smith’s [1] original enquiry. An understanding of the main determinants of long-run growth arguably remains the most important issue in economics.

The ultimate causes of economic growth are however still not fully understood. In an influential study, Helpman [2] examined the recent literature on the subject and acknowledged that the determinants of economic growth remain a mystery. Early models based on capital deepening and exogenous technical progress have given way to contributions that emphasise the endogenous nature of economic growth and that explore the role of factors such as expenditure on education, investment in research and development, openness to international trade and the presence of institutions that foster social and economic inclusion [3,4,5].

Whilst all of these factors play a role in affecting economic growth, the relative importance of each of them is more difficult to establish, especially because they are bound to interact with each other in complex ways. Furthermore, this list of key determinants of economic growth may not be exhaustive: additional factors that have hitherto been ignored may be just as important and may contribute significant predictive power to the models that have been studied so far. Exogenous, as well as endogenous growth theories predict that growth can be explained by a set of variables that capture both the initial conditions of the economy and the rate at which its production inputs are accumulated. The extent to which these models can predict the growth performance of broad cross-sections of countries or regions is however limited [5]. It is therefore important to look for additional drivers of growth, which may have been ignored in the more traditional analyses.

The present paper explores one such additional factor: the fitness of the economy, as measured by a complex-network metric based on the countries’ revealed comparative advantage [6], the Economic Fitness-Complexity algorithm (EFC). The profile of trade specialization of a country can in fact be regarded as a reflection of its underlying capabilities, which are defined as the skills that enable its economy to expand into new production requirements and to adopt new technologies [7]. Conventional measures of the production possibilities of an economy do not explicitly consider its degree of flexibility and adaptability: if more complex economies are however also endowed with a richer set of capabilities, then ignoring them would lead to a misspecification of the underlying models, because relevant explanatory variables would be omitted from the analysis.

The structure of this paper is as follows. Section 2 discusses the role of the capabilities and complexity for economic growth. Section 3 defines the measure of fitness used in the paper and explains the empirical methodology and the data. Section 4 presents the empirical evidence obtained with a non-parametric graphical analysis. Section 5 concludes.

## 2. Economic Growth, Capabilities and Complexity

In his encyclopaedic treatment of modern theories of economic growth, Acemoglu [4] offered a thorough overview of the main approaches that have been set out to explain the dynamic growth paths of the economies in the long run. The early analysis by Solow [8] identified the main sources of growth in the accumulation of physical capital and in exogenous technical progress, which is responsible for the upward shift of the production possibility frontier of the economy over time. In the presence of decreasing returns to capital in the aggregate production function, Solow’s model predicts that the rate of growth will eventually tend to peter out, as the economy approaches its long-run steady-state dynamic equilibrium path. An important implication of the exogenous growth model is that the income per capita of low-income economies should tend to converge to that of high-income ones in the long run, and we should therefore observe a catching-up of less developed economies to the more developed ones. Empirical analyses show that there is some evidence of the convergence of low income to high income economies, albeit at a very slow pace [5].

More recent analyses of growth have sought to endogenize the rate of technical progress as the outcome of explicit decisions made by economic agents. These models overcome the assumption of decreasing returns with respect to the factor that is accumulated by acknowledging the existence of externalities among firms. This view was first explored by Arrow [9] and is captured in his notion of learning-by-doing. New technologies are incorporated in new investments, and therefore, the rate at which aggregate productivity increases is directly related to the rate of new investment in the economy. As a result, the productivity of individual firms is an increasing function of the aggregate capital stock in the economy. This way, it is possible to have constant or even increasing returns to capital accumulation at the aggregate level, even if the technology at the firm level still exhibits conventional decreasing returns to its own production inputs, as shown by Romer [10,11].

Later developments in endogenous growth theories identify the main drivers of long-run growth in investment in education in the presence of externalities from human capital accumulation [12], expenditure in research and development in a stochastic Schumpeterian model of creative destruction [13] or the openness of the economy to international trade through learning-by-exporting [14,15]. These drivers may interact with each other, as in the model by Lucas [16], where a high level of human capital in the economy facilitates investment in new technologies, and in turn, this enhances their growth-improving effects. The recent literature on growth has also explored the role of social and political institutions. In particular, Acemoglu and Robinson [17] distinguished between inclusive institutions, which stimulate entrepreneurship and innovations, and extractive institutions, which are instead responsible for creating incentives to exploit rents and which have a dampening effect on growth.

Endogenous growth theories are able to explain the lack of convergence of poorer countries and regions to the richer ones: since the returns to the factors that are accumulated are now constant or even increasing, the rate of growth of the economy is not necessarily predicted to slow down along its long-run equilibrium path. A further important extension of growth models considers the possibility of multiple steady state equilibria. Murphy, Shleifer and Vishny [18] formalise the notion of a big push, according to which a discrete development effort is required for a low-income economy to escape its poverty trap and to set in motion a process of self-sustaining growth. The rationale for multiple equilibria rests on the assumptions of increasing returns in the scale of production and of non-pecuniary externalities among sectors. Multiple equilibria can provide a justification for targeted development policies, for instance in the form of government support to industrialisation in a critical mass of industrial sectors. Models with multiple steady-state equilibria have been generalised to models with multiple dynamic steady states (e.g., Krugman [19] and Matsuyama [20]), where initial conditions can be critical to determine the long-run growth path of the economy.

A novel approach to the analysis of economic growth, however, goes beyond aggregate measures of the production inputs and considers instead a more granular and structural view of the production possibilities of the economy. This approach examines the possible role of capabilities, which could be defined as a broad set of skills that could adapt to changing production requirements and which facilitate the introduction of new technologies. This approach can be traced back to the seminal work by Hirschman [21], where capabilities make it possible to create backward and forward linkages across economic sectors, and Penrose [22], with her resource-based theory of the firm. Teece et al. [23] argued that capabilities in an organization are both intangible and non-transferable, and laid the foundations for the modern complex network analysis by showing that they constitute the key factors for a coherent growth of the firm consistent with its core competencies. Abramovitz [24] referred to social capabilities as all those attributes that affect a country’s ability to operate modern and large-scale businesses and which include their political and social characteristics: they should therefore not be interpreted in a narrow individualistic sense (see also Fagerberg and Srholec [25]). Capabilities can affect economic growth directly through their impact on the adoption of innovations, as shown by Lall [26] and by Kremer [27].

In a series of important contributions, Sutton [28,29] and Sutton and Trefler [30] argued that the source of the main differences in output per capita across economies lies not in their accumulation of physical factors of production, as in the conventional theories of growth, but rather in the set of capabilities with which their economy is endowed. These capabilities enable firms to take advantage of investment by increasing their labour productivity and also make it possible to further expand their set of skills, thereby generating a virtuous circle. Sutton’s analysis combines insights from the industrial organization literature, the economic geography literature and the trade literature. Central to his results is that, in equilibrium, the market structure of an industry will tend to encompass a variety of products of different quality. The capabilities required to produce high-quality products will always be scarce. This implies that, on a global scale, in each industry, there will only be a limited number of firms that hold a dominant position. From economic geography, capabilities must be concentrated in some countries in order to take full advantage of agglomeration economies. Each country’s comparative advantage will therefore reflect what Sutton calls the “economics of quality”. The available capabilities become a key factor in global trade specialization and in determining the growth prospects of each country.

Capabilities are however unobservable. Hausmann, Hwang and Rodrik [31] argued that they can be inferred from a country’s export specialization profile. Production of complex goods involves the joint execution of a large number of highly specialised tasks [27,32]. This requires both the existence of broad sets of advanced skills and the ability to combine them effectively. A detailed examination of the revealed comparative advantage across all countries in the world using the tools of complex network analysis can identify those countries that specialise in complex products. Their production could involve a number of tasks by high-skilled workers, who could not be substituted by low-skill workers without compromising the quality of the finished product [27]. The sophisticated production structure of these countries also allows them more easily to introduce innovations and to adopt advanced technologies consistent with the role of dynamic capabilities in the environment of rapid technological change analysed by Teece et al. [7]. The location of a country in the global product space would therefore be a significant predictor of its growth potential [33,34].

The usefulness of measures of product complexity for predicting growth has been demonstrated by Hausmann, Hwang and Rodrik [31], who show that the mix of good that a country produces can explain its growth rate. Ferrarini and Scaramozzino [35] confirmed that production complexity can help explain differences in economic performance, in an endogenous growth model with human capital accumulation. Pugliese et al. [36] analysed the role of economic fitness during the process of development and industrialization showing that fitness crucially eases the passage towards sustained growth. Sbardella et al. [37] empirically demonstrated that fitness is a key variable in explaining the relationship between wage inequality and economic development. Boltho et al. [38] argued that the more complex production structure in East Germany relative to that in the Italian Mezzogiorno can contribute to explaining the unsatisfactory performance of the latter and its lack of convergence to the more prosperous regions in the Centre-North of the country. Zaccaria et al. [39] discussed the application of such an economic complexity approach to the analysis of the production structure of the Netherlands; in particular, they studied the time evolution of the competitiveness and the complexity of strategic industrial sectors. In a recent World Bank technical report, Zaccaria et al. [40] studied how fitness assessments are affected when trade in services is included in the data fed to the EFC algorithm.

Cristelli et al. [41,42] developed an innovative approach to predict economic growth, the Selective Predictability Scheme (SPS), which makes use of ideas from the theory of economic complexity and which adopts the economic fitness metric. SPS is shown to out-perform a number of alternative forecasting models, thus confirming the key role played by the complexity of the structure of production for predicting the future economic performance of a country. Tacchella et al. [43] further developed this approach to growth forecasting and out-performed the accuracy of the IMF five-year forecasts by more than 25%.

## 3. Materials and Methods

### 3.1. Measuring Fitness

As mentioned above, in this paper, we examine the complexity-economic growth nexus. In order to do so, we employ the Economic Fitness-Complexity approach (EFC) [6,44,45]. Fitness is a measure of economic complexity, i.e., a proxy for capabilities, based on cross-country differences in productive and export structures. In fact, following the points made in Section 2, GDP may not be a sufficient statistic to describe the development and growth processes, as two countries with similar GDP levels may actually possess profoundly different endowments of capabilities. Countries that are below the income expected from their economic performances may have already developed the full range of products that is within their technological reach; nevertheless, this capability level may have not been yet translated into higher GDP levels. In this perspective, different endowments of capabilities are the main, albeit not empirically observable, sources in explaining different economic performances and in shaping the export profiles of countries.

**Figure 1:**
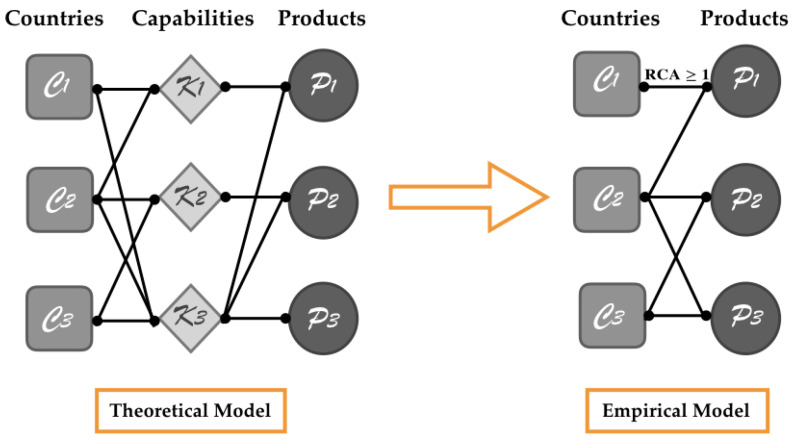
In our theoretical framework, capabilities are crucial in explaining the economic performances of countries. As can be observed on the left, conceptually, we can visualize capabilities as an intermediate layer in a tripartite country-capability-product network. Capabilities are non-measurable entities, and information on them can be inferred by building an empirical country-product network through international trade data. As can be seen on the right, such a bipartite network can be interpreted as the projection of the tripartite network. In the country-product network, a C→P link is established if and only if the country has a revealed comparative advantage in exporting the product [44,45].

In network theory, this notion of capabilities can be conceptually represented as an intermediate network layer that connects countries to their exported products [44,45,46]. As schematically illustrated in Figure 1, it is possible to define a tripartite network whose three classes of nodes are countries (C), capabilities (K) and products (P). The links, connecting the K nodes to the C or P nodes, describe the capability owned by a country (C→K links) and the capability required to export a product with a comparative advantage (K→P links). By defining an ad hoc toy model, Tacchella et al. [44] explored the functioning of this tripartite network and showed the high degree of correlation between a country’s endowment of capabilities and its fitness in a simplified world of 120 countries, 200 capabilities and 800 products. However, the capability layer, despite being conceptually crucial, is intangible, and the tripartite network is a purely theoretical tool to better visualize our framework. Relying on international trade data, the information coded in the hidden capability layer can be gathered by building an empirical bipartite network in which countries are connected to the products they export [44,45,46] with a Revealed Comparative Advantage (RCA) [47]. As represented in Figure 1, this country-product network is viewed as a contraction of the tripartite network over the capability dimension.

**Figure 2:**
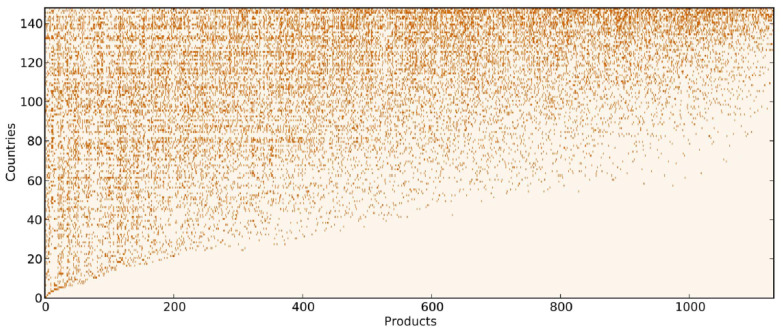
The binary matrix of countries and products built from the worldwide 1998 export flows of the BACI dataset [48]. The rows and columns of the matrix are ranked according to the Economic Fitness-Complexity algorithm (EFC). The rows are sorted by increasing country fitness and the columns by increasing product complexity. In such a way, the matrix acquires a triangular-like shape: countries with more diversified export baskets are more competitive, while countries specialized in a few products—which generally are also exported by every other country—are the less competitive. Source of the figure: Tacchella et al. [44]. With the permission of Publisher Elsevier.

How does one extract in an optimal way the informative content coded in such a country-product network? The fitness-complexity metric provides the correct mathematical specification based on the network topology, and through an iterative algorithm, it defines a measure of country competitiveness, i.e., fitness, and of product sophistication, i.e., complexity. To understand the rationale beyond the metric, it is useful to observe the binary adjacency matrix of the network, $\hat{M}$, whose rows represent countries and columns their exported products (see Figure 2). RCA is used to filter and digitize the data allowing one to focus on qualitative differences, rather than quantitative, in the export baskets of countries: which kinds of products are exported competitively, not in which volumes. Therefore, matrix entries are set equal to one when a country exports a product with an RCA greater than or equal to one, and zero in the opposite case:

(1) $$\begin{array}{c} M_{cp}=\left\{\begin{array}{cc} 1 & \mathrm{if}\;RCA_{cp}\geq 1\\ 0 & \mathrm{if}\;RCA_{cp}<1. \end{array}\right. \end{array}$$

If suitably ordered, the matrix $\hat{M}$ assumes a quasi-triangular shape. Indeed, by looking at the matrix triangularity, it is possible to infer that, on the one hand, a largely diversified country’s ability to export a product with a comparative advantage gives no clues about its complexity. On the other hand, when a poorly-diversified country—which has an RCA in a few and very ubiquitous goods—is able to export a product with a comparative advantage, it is likely that its manufacture requires a low level of sophistication. This is quite informative: a product is complex if low-fitness countries do not export it. To make this clearer, consider a straightforward example of a high complexity product, transistors, and a low complexity product, nails. Only highly industrially- and technologically-developed countries are able to export transistors; by contrast, nails are exported by all sorts of countries, both more and less industrialized. Consequently, the low complexity of nails can be surmised directly from their presence in the export basket of low fitness countries. This observation hints at a non-linearity in the relation between product complexity and country fitness. The need for a non-linear coupling between fitness and complexity was formalized by Caldarelli et al. [49]. From the matrix $\hat{M}$, thus, it is possible to obtain an intensive metric that measures country fitness ($F_{c}$) as a weighted diversification of the country export basket, where the weight is the complexity associated with each product. Product complexity ($Q_{p}$), instead, is calculated as the number of countries that export the product with comparative advantage, bounded by the fitness of the least competitive exporter of the product. In mathematical terms, the Economic Fitness-Complexity algorithm (EFC) is defined as:

(2) $$\begin{array}{c} \left\{\begin{array}{c} {\tilde{F}}_{c}^{\left(n\right)}=\sum_{p}M_{cp}Q_{p}^{\left(n-1\right)}\\ {\tilde{Q}}_{p}^{\left(n\right)}=\frac{1}{\sum_{c}M_{cp}\frac{1}{F_{c}^{\left(n\right)}}} \end{array}\right.\left\{\begin{array}{c} F_{c}^{\left(n\right)}=\frac{{\tilde{F}}_{c}^{\left(n\right)}}{<{\tilde{F}}_{c}^{\left(n\right)}{>}_{c}}\\ Q_{p}^{\left(n\right)}=\frac{{\tilde{Q}}_{p}^{\left(n\right)}}{<{\tilde{Q}}_{p}^{\left(n\right)}{>}_{p}} \end{array}\right. \end{array}$$

where $<\cdot {>}_{x}$ denotes the arithmetic mean with respect to the possible values assumed by the variable dependent on *x*, with the initial condition:

(3) $$\sum_{p}Q_{p}^{\left(0\right)}=1\;\forall \;p.$$

The iteration of the coupled equations leads to a fixed point, which has been proven to be stable and non-dependent on initial conditions [6]. The fixed point defines the non-monetary metric, which actually quantifies $F_{c}$ and $Q_{p}$. The convergence properties of the algorithm defined in Equation (2) are not trivial and have been extensively studied by Pugliese et al. [50].

### 3.2. Empirical Strategy

As discussed in the previous sections, the economic complexity approach has a structural interpretation in terms of growth and development, understood as the outcomes of a learning process through which new capabilities are added to the existing pool, thus opening up new and more complex productive possibilities, which will eventually lead to higher prosperity and faster economic growth.

In this section, to partially reconcile this novel view on development with the stylized facts of neoclassical theories of economic growth, we integrate the economic complexity discourse with the analyses of growth determinants popularized in the 1990s by Barro [51] and Mankiw, Romer and Weil [52], the so-called “growth regression models”. In the latter, GDP per capita growth is decomposed into contributions associated with changes in factor inputs, production technologies, demographic variables, and so on. In particular, here we focus on the multifaceted relations between GDP per capita growth, economic fitness and various economic indicators considered crucial drivers for economic growth: GDP per capita, capital intensity, employment ratio, life expectancy, human capital and total factor productivity. The choice of such indicators is rooted in the growth regression models; notably, the income- and capital-deepening variables are directly linked to the early analysis of Solow [8], while the inclusion of data on educational attainment and life expectancy among the explanatory variables is based on the more recent endogenous growth theories mentioned in Section 2.

We follow the lines of Cristelli et al. who argue that a parametric approach might fail to capture the highly heterogeneous patterns of evolution of countries that they observe in the fitness-GDP plane, based on which they define different economic phases for the predictability of growth by using the tools of dynamical system theory. In fact, a parametric approach, looking at averaged interactions and having strong assumptions on the functional forms of the relationships, might fail to recognise the importance of a strong, but non-linear signal. For instance, a discrete element as the presence or absence of an enabling capability necessary to export a product competitively that can change the development profile of a country and interact with the other variables in unpredictable ways might remain undetected when using a parametric approach. On this basis, we opt for a non-parametric description since the complex interactions between fitness, the chosen growth determinants and economic growth are fundamentally dynamic and non-linear, possibly changing their nature depending on the phase that an economy is going through: in each phase, the dimensions of interest recombine in an a priori unknown fashion.

**Figure A1:**
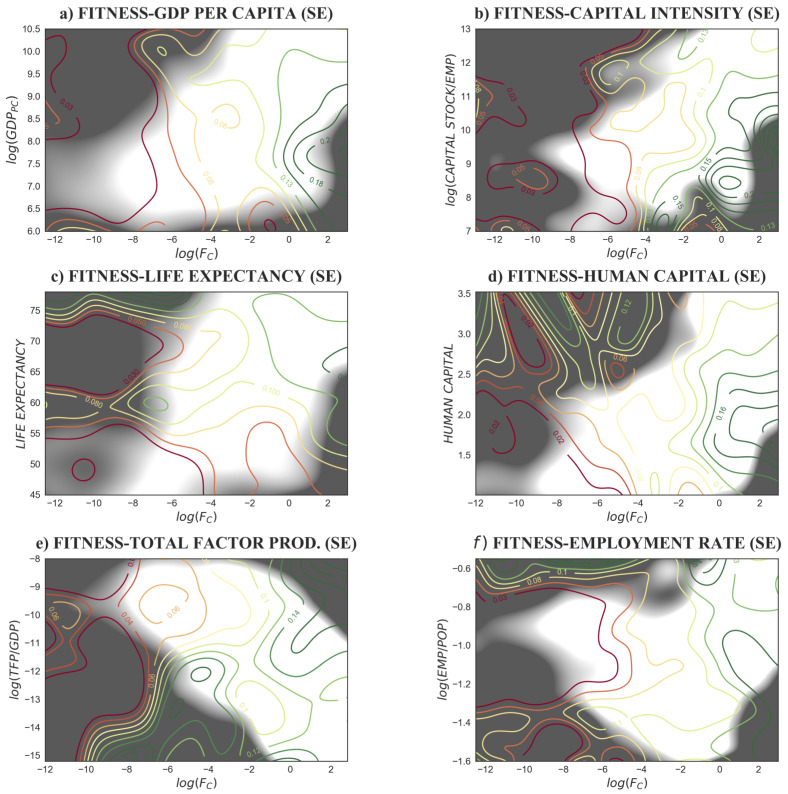
Estimation errors of the growth rate colour-maps in Section 4. Two layers of information are represented in the figures. (1) In the grey scale, the Nadaraya–Watson growth rate estimation error. White indicates a standard error of 0.2% or less and dark grey a standard error of 0.4% or more. (2) The iso-lines of the growth rate levels, represented in the same palette of the colour maps (lowest in dark red, highest in dark green).

In practice, we explore these empirical relations by looking at tridimensional colour-maps, smoothed representations of GDP per capita growth rate for different values of fitness on the *x*-axis and, on the *y*-axis, of each one of GDP per capita, capital intensity, employment ratio, life expectancy, human capital and total factor productivity. Our empirical evidence is obtained by using an unbalanced panel of countries over the time period 1963–2000, the number of countries slightly varying around 144. In order to avoid cyclical short-run fluctuations, the GDP per capita growth rate is calculated over Δt=5 years. Furthermore, to reduce the risk of simultaneity bias, a lag of five years is considered between the explanatory variables and GDP per capita growth rate. The colour-maps are realized through a multivariate Nadaraya–Watson regression [53], a continuous non-parametric kernel estimator, and a time lag Δt=5 years is considered between the x,y variables and the growth rate on the *z*-axis. Notice that the standard errors of the following growth rate non-parametric estimations can be found in Figure A1 of Appendix A, where, to allow for comparability with the figures in the main text, the iso-levels of the growth rate are superimposed on the sub-plots.

### 3.3. Sources of Data

The levels of GDP, population, total factor productivity, capital endowments and human capital (proxied by a measure of educational attainment and returns to education) are taken from the Penn World Table 9.0 (PWT) produced by the University of Groningen and the University of Pennsylvania [54]. The PWT dataset covers 167 countries over the period 1950–2011.

We also include in our analysis the Life Expectancy measure of the World Development Indicators, the World Bank collection of development statistics [55]. The data are provided from 1960–2016 and cover more than 200 countries.

Finally, fitness is taken from the export fitness dataset developed by the PIL group of the Institute of Complex Systems-CNR (Rome), which covers a number of countries varying between 130 and 151, over the period 1963–2000 [36]. As briefly explained in the previous section and shown in detail in Tacchella et al. [6,44] and Cristelli et al. [45], fitness is obtained through an empirical algorithm that, for the chosen time window, employs international trade data from UN-COMTRADE [56]. The choice of the time period of our analysis was constrained by the availability of fitness data.

Table 1 lists and describes all the variables as they are used in our empirical analysis, also reporting their means, medians and standard deviations to convey a sense of their magnitudes.

## 4. Empirical Evidence

### 4.1. Fitness and GDP per capita

As follows from the hypothesis of diminishing returns with respect to the factor which is accumulated, exogenous growth theories predict higher growth in response to lower starting GDP per capita, since in the long-run, all economies should converge to their steady-state income levels. Initial GDP per capita, thus, should have a negative impact on economic growth and low-income countries should tend to grow at faster rates. Such conditional convergence has been reported, even if not to the extent predicted by neoclassical models, by Barro [57] and Mankiw et al. [52]. However, other important empirical contributions do not find evidence of it [58,59].

**Figure 3:**
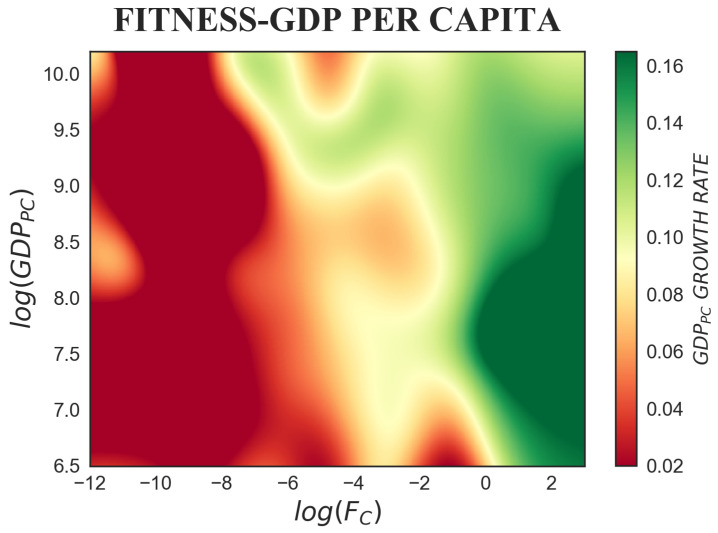
The colour-map represents the tridimensional relation between fitness, GDP per capita and subsequent GDP per capita growth rate, where Δt=5 years is considered. Countries with low fitness are not able to achieve subsequent high growth rates, irrespective of their initial GDP per capita level. For countries with intermediate fitness, higher GDP per capita results in mildly higher growth rates. Countries with high fitness are able to grow at very high rates, especially when their GDP per capita is low or intermediate. Fitness, when it is put into relation with GDP per capita, is able to suggest future growth in a way not fully captured by the sole information contained in the GDP per capita.

The predominantly diagonal variability of colour in Figure 3 shows that fitness is decisive in determining the growth profile of countries [36,41,42]. By looking at the empirical colour-map, we can distinguish three regions. First, in the case of countries with low initial fitness ($-12<log(F_{c})⩽-7$), GDP per capita growth rate is completely led by fitness. When fitness is too low, independent of the initial income level, the take-off does not occur. A critical degree of complexity is necessary to start the catch-up [36]. Second, in the portion of the plot with countries that exhibit intermediate fitness ($-7<log(F_{c})<0$), contrary to the predictions of exogenous growth theory but consistent with the endogenous ones, higher starting GDP per capita results in mildly higher growth rates. Here, for example, we find countries whose high income is mainly driven by oil exports and does not reflect the endowment of production capabilities that would be necessary to sustain high economic growth. Third, the convergence hypothesis applies to high fitness countries ($log(F_{c})⩾0$). Solow’s model describes the technological possibilities of a country solely in terms of its production function. It is therefore not surprising that its predictions do not fully capture the role also played by capabilities and are meaningful only for countries that possess the capabilities to produce most products.

This brings to light a complementarity between the role of the two indicators: countries with low GDP per capita but high fitness have large capability portfolios, which, after a critical threshold, enable them to catch-up and achieve very fast growth rates. As has been pointed out by Cristelli et al. and Pugliese et al., in this region of the fitness-GDP per capita plane, we can find, among others, China and South Korea. Moreover, in Cristelli et al. [41] and in different other recent contributions [42,43], it has been explored in depth how fitness, when put into relation with GDP per capita, is a very powerful instrument for predicting the growth potential of countries. Indeed, fitness, being a structural measure that quantifies capability endowments, is able to profile the growth potential of countries in a more nuanced and coarse-grained way relative to income per capita and other aggregate measures of growth inputs.

### 4.2. Fitness and Capital Intensity

Capital intensity is defined as the amount of physical capital in relation to labour, i.e., the ratio between physical capital stock and the number of workers employed in the economy. Under the neoclassical convergence hypothesis, in the long-run, capital-intensive societies should tend to have higher standards of living, with higher GDP per capita levels and lower growth rates. By contrast, emerging economies should tend to be labour intensive and show higher growth rates. Accordingly, from the bottom to the top of our colour-map, we should observe that as capital intensity increases, the colour should go from green (higher growth rate) to red (lower growth rate).

**Figure 4:**
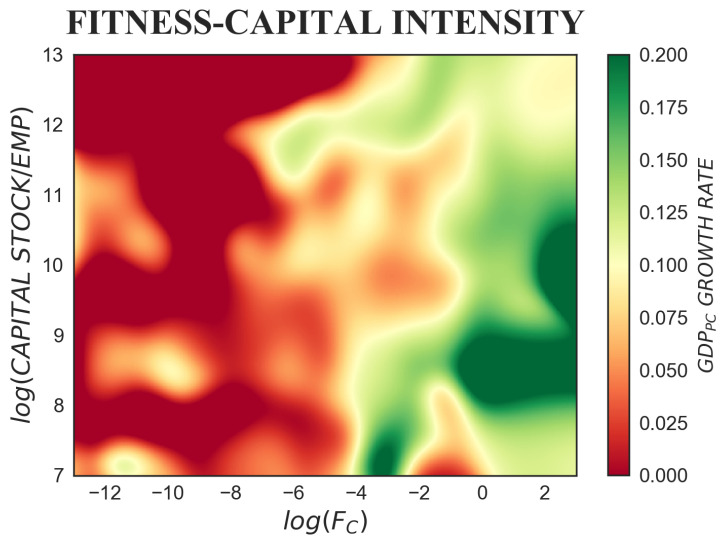
The colour-map represents the tridimensional relation between fitness, capital intensity and subsequent GDP per capita growth rate, where Δt=5 years is considered. When the combined effect of fitness and capital intensity is taken into consideration, the latter loses explanatory power, and the growth profile of countries is almost completely explained by their fitness level. Higher fitness leads to higher growth rates, and countries with high fitness and intermediate capital intensity are able to achieve the highest growth rates.

However, in Figure 4, when the combined effect of fitness and capital intensity is taken into consideration, K/EMP loses explanatory power with respect to fitness. The movement of colour is mostly horizontal and goes from red to green: keeping K/EMP on the *y*-axis constant, the growth rate rises as fitness increases on the *x*-axis. Therefore, higher fitness can compensate lower capital endowments, and fitness can contribute in keeping high growth rates even in the presence of low values of K/EMP. Nevertheless, a partial agreement with the neoclassical predictions is found for very high starting fitness countries ($log(F_{C})>0.5$). The latter, similarly to what was examined in Figure 3 focusing on income per capita, are able to achieve faster growth rates with low or intermediate capital intensity.

### 4.3. Fitness and Employment Ratio

**Figure 5:**
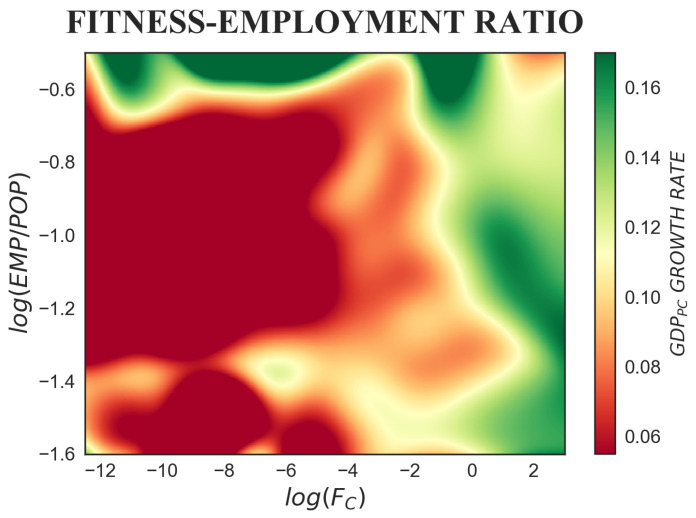
The colour-map represents the tridimensional relation between fitness, employment ratio and subsequent GDP per capita growth rate, where Δt=5 years is considered. Only the highest levels of employment ratio, after a critical threshold of log(EMP/POP)∼−0.65%, have a positive effect on economic growth. This is clearly visible from the horizontal variation of colour from red to green in the upper portion of the plot. For lower values of EMP/POP, the dominant variation of the colour is vertical: the higher the fitness, the higher the growth rate.

The employment ratio is here defined as the employment-to-population ratio, where employment levels include all the persons working within national boundaries. According to endogenous growth models, the employment ratio should have a positive effect on economic growth; in particular, Arrow’s learning-by-doing model [9] predicts that higher employment ratios will create positive externalities due to a larger size of the market. In Figure 5, the colour progression hints at the presence of non-linear dependencies, and different behaviours of GDP per capita growth rate can be identified. The predicted positive effect of the employment ratio on economic growth is detected only for the highest levels of employment ratio, after a critical threshold of log(EMP/POP)∼−0.65% (EMP/POP∼52%), as can be seen from the horizontal variation of colour from red to green in the left-upper portion of the plot. By contrast, for lower values of the employment ratio, fitness acquires higher explanatory power and the dominant variation of the colour is vertical.

### 4.4. Fitness and Life Expectancy

**Figure 6:**
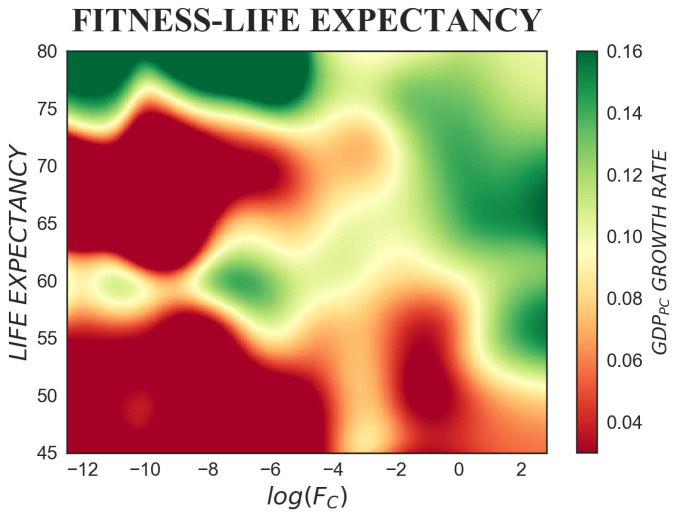
The colour-map represents the tridimensional relation between fitness, life expectancy and subsequent GDP per capita growth rate, where Δt=5 years is considered. Life expectancy values ≳73 years have a positive effect on growth rates. When life expectancy <73 years, fitness determines the colour contour: the higher the fitness, the higher the growth rate. However, also high fitness countries show a higher growth rate when life expectancy >60 years.

Life expectancy at birth (measured in years of life) is a proxy for healthcare quality. According to the growth regressions by Barro [5,51], the effect of life expectancy on economic outcomes is positive and significant: better health should lead to higher economic growth. In Figure 6, such a positive effect is only obtained when a critical value of circa 73 years is exceeded. Similarly to Figure 5, here too, a non-linear behaviour is found, and fitness almost entirely dominates the dynamics. For lower values of life expectancy, low fitness countries grow slowly, whilst the opposite holds for high fitness countries. Nevertheless, also for high fitness countries, growth rates are higher if life expectancy is approximately higher than 60 years. Such a threshold falls for very high fitness countries ($log(F_{C})>0.5$): lower life expectancy is offset by increasing capabilities.

However, when it comes to demographic variables, setting the direction of the arrow of causality may pose some problems. Life expectancy is listed among the growth determinants, but also the opposite relation is likely, since high growth rates might lift up life expectancy.

### 4.5. Fitness and Human Capital

As mentioned, here human capital is proxied by a measure of educational attainment and returns to education. In endogenous growth theories, human capital is an essential ingredient to achieve sustained growth rates. The accumulation of human capital, through formal training or learning-by-doing, creates positive externalities through increased productivity and technological innovation originating from the process [12]. In Barro’s growth regressions [5,51], human capital is positively and significantly related to subsequent GDP per capita growth rates.

**Figure 7:**
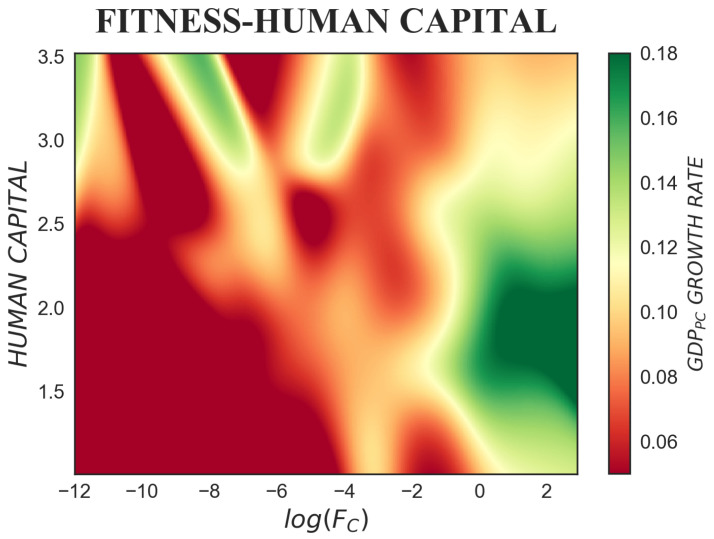
The colour-map represents the tridimensional relation between fitness, human capital and subsequent GDP per capita growth rate, where Δt=5 years is considered. Fitness and human capital appear positively correlated in some regions of the plots, and complementary in others. Low and intermediate fitness corresponds to low human capital. While, when $log(F_{c})>-4$, increasing fitness affects positively the GDP per capita growth rate, even for low human capital levels.

Figure 7 puts into light a positive relation between fitness and human capital, principally for intermediate and high fitness values. In some regions of the plane, fitness and human capital are entangled and reinforce each other, while in others, high initial fitness can compensate for a lack of initial human capital. Low and intermediate fitness countries ($log(F_{c})\leq -4$) are positively associated with low human capital (<2). By contrast, when $log(F_{c})>-4$, increasing fitness has a positive effect on economic growth even if human capital is low. For $log(F_{c})>-2$, human capital takes off, and the fitness-human capital relation starts to assume an increasing shape. In this portion of the plot, we partially recover the expected effect of human capital on economic growth; fitness, though, still maintains a leading position. Notably, two main features of the relationships can be highlighted. First, as expected, countries with very high human capital levels grow slowly, as can be seen in the upper right corner of the figure. Second, in the bottom right corner are placed countries that, having very high fitness, are able to enter into sustained growth regimes even with low human capital. In such a way—as can be appreciated by the vertical passage from red to green, and then back to red—for high and intermediate fitness countries, we observe an inverted U-shaped relationship between human capital and the growth rate. Low human capital predicts a low growth rate (lower red-yellow area); intermediate human capital predicts high growth rates (intense green area); and finally, when human capital is high, the subsequent growth rates are low (upper orange area), but not as in the first phase.

### 4.6. Fitness and Total Factor Productivity

In the neoclassical production function, Total Factor Productivity (TFP)—or the Solow residual—is identified as the growth of output that cannot be accounted for by the growth of the observed inputs, capital and labour. According to exogenous growth theories, TFP indirectly captures technological improvements (i.e., productivity growth), and in this framework, an increase in total factor productivity should shift the growth rate upwards.

**Figure 8:**
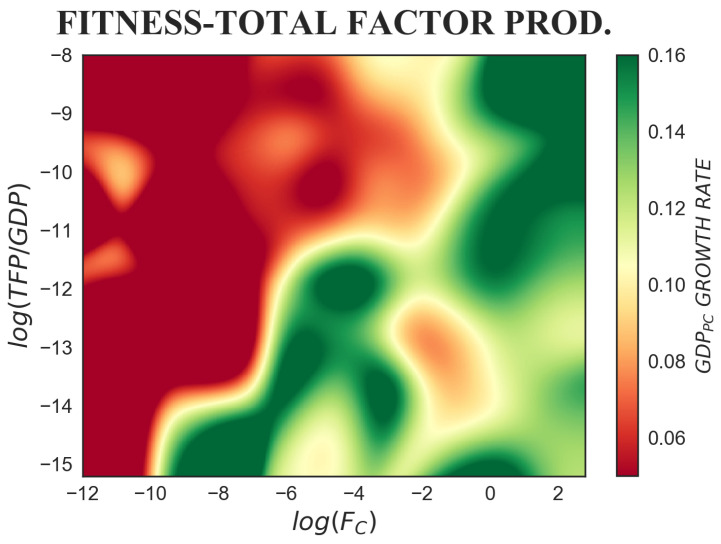
The colour-map represents the tridimensional relation between fitness, total factor productivity and subsequent GDP per capita growth rate, where Δt=5 years is considered. For log(TFP/GDP)≳−11, as can be noted by the horizontal movement of colour from red to green, fitness is the prevailing factor. Keeping Total Factor Productivity constant, high fitness corresponds to greater growth rates. For log(TFP/GDP)<−11 from the diagonal variation of colour, we deduce that fitness and TFP are complementary in affecting future growth rates. In this area, very low fitness brings low growth, independent of the TFP level. For low and intermediate fitness countries, TFP has a negative impact on economic growth. This could be due to TFP measurement errors. Finally, we detect a complementarity between fitness and TFP for highly competitive economies: high fitness leads to high growth rates, especially when TFP is high.

However, as can be noted by the horizontal movement of colour from red to green, on the one hand, in the upper portion of Figure 8, for log(TFP/GDP)≳−11, the dynamics is dominated by fitness. Keeping TFP constant, the higher the fitness, the greater the growth rate. On the other hand, for log(TFP/GDP)<−11, as is apparent from the diagonal variation of colour, even if fitness remains the prevailing factor, a complementarity between its effect and that of total factor productivity slowly emerges. This region can be divided into three according to the corresponding fitness level. First, for very low fitness ($-12<log(F_{c})<-10$), we observe low growth rates, irrespective of the level of total factor productivity. Second, for low and intermediate fitness ($-10<log(F_{c})<-2$), we observe a negative effect of total factor productivity on economic growth. Indeed, since TFP is estimated as a residual in the decomposition of the growth rate, all the measurement errors in capital and labour will be reflected in total factor productivity. Thus, our finding might be attributed to measurement errors in TFP, possibly due to an overestimation of the labour and capital factors. The source of such overestimation might be a misattribution of capabilities to capital or labour. Third, for $log(F_{c})>-2$, growth rates are generally higher. However, the vertical transition of colour from yellow to green shows that, as suggested by Solow’s theory, total factor productivity positively affects the growth rate. Therefore, in this region of the plane, fitness and TFP are mutually reinforcing.

## 5. Conclusions

This paper builds a bridge between the economic complexity and the economic growth literature, and through a non-parametric analysis, it shows that complex network theory can be a powerful tool to understand the process of growth. The traditional literature on growth does not fully acknowledge the role played by the underlying production capabilities with which an economy is endowed. These are however crucial for the early stage of the industrialization process, as well as for the adoption of advanced technologies and for the introduction of innovations. The fitness metric used in the paper, which relies on the global export specialization profile of countries to infer their underlying production capabilities, proves to be a significant variable for predicting growth. The non-parametric kernel regressions, which do not assume any a priori functional forms for the relationships, show the combined effect of fitness and of other key determinants of growth: GDP per capita, capital intensity, employment ratio, life expectancy, human capital and total factor productivity. Our findings are unambiguous. Fitness is an important, if not the most important, player in enhancing economic growth. Depending on the drivers and on the different phase that an economy is going through, when fitness is included in the analysis, it can be either complementary to traditional drivers of growth or can completely overshadow them. Complementarity emerges in particular for high-fitness countries, which already possess a rich basket of underlying capabilities and are able to be competitive in a huge variety of products. As a consequence, since describing the technological possibilities of countries solely in terms of their production functions disregards the role of capabilities, for such high-fitness economies, the observed relationships between the chosen variables and the growth rate are still in agreement with the neoclassical and endogenous growth predictions. By contrast, when capabilities are a constraint, unexpected behaviours emerge. The growth rate of low and intermediate fitness countries, whose capabilities are quite heterogeneous, appears to be mostly driven by fitness. We can thus conclude that different endowments of capabilities explain differences in economic performances, since greater complexity enables countries to use factors more efficiently, be more competitive and grow more rapidly [28,29,30,36,42]. This suggests that, once capabilities are accounted for, other factors lose importance. Economic models of growth that overlook the role of complexity therefore risk being severely misspecified.

## Figures and Tables

**Table 1:** Definitions, sources, means, medians and standard deviations of variables.

Used Variable	Mean	Median	St. Dev.	Source & Variable Names	Description
$GDP_{pc}$ Growth Rate	0.08	0.09	0.18	PWT 9 Penn World Tables 9.0 [54] (based on rgdpna and pop)	Annual growth rate of GDP per capita (rgdpna at constant 2011 national prices in 2011 U.S. $ million, pop in millions). In levels, formula: $log\left(GDP_{pc}\left(y\right)\right)-log\left(GDP_{pc}\left(y-5\right)\right)$.
EMP/POP	−1.03	−1.00	0.26	PWT 9 Penn World Tables 9.0 [54] (emp/pop)	Employment ratio (emp in millions, pop in millions). In natural logarithm, at year y+5.
GDP/POP	8.54	8.56	1.17	PWT 9 Penn World Tables 9.0 [54] (rgdpo/pop)	Income per capita (rgdpo in 2011 U.S. $ millions, pop in millions). In natural logarithm, at year y+5.
K/EMP	10.16	10.31	1.52	PWT 9 Penn World Tables 9.0 [54] (ck/emp)	Capital intensity (ck in 2011 U.S. $ million, emp in millions). In natural logarithm, at year y+5.
HUMANCAPITAL	1.94	1.82	0.66	PWT 9 Penn World Tables 9.0 [54] (hc)	Human capital (human capital index, based on years of schooling and returns to education, adimensional). In levels, at year y+5.
TFP/GDP	−11.46	−11.53	1.79	PWT 9 Penn World Tables 9.0 [54] (rtfpna/rgdpna)	Total factor productivity-GDP ratio (rtfpna at constant national prices with 2011 = 1, rgdpna at constant 2011 national prices in 2011 U.S. $ million). In natural logarithm, at year y+5.
LIFEEXPECTANCY	62.92	66.18	10.97	WDI World Development Indicators, The World Bank Group [55] (Life expectancy at birth, total)	Life expectancy at birth (in years of life). In levels, at year y+5.
FITNESS	−2.39	−1.16	4.37	PIL-FF 1963-2000 [36] (*fitness scores*)	Fitness (PIL’s group computation from EFC algorithm [6] based on UN-COMTRADEexport data [56], adimensional). In natural logarithm, at year y+5.

## Appendix A. Non-Parametric Graphical Analysis: Estimation Errors

To validate the non-parametric estimations of the relationship between the growth rate, fitness and the chosen growth drivers in Section 4, in Figure A1, we represent the Standard Error (SE) of the Nadaraya–Watson estimations of the growth rate.

The plots are built with the same data of the figures in the main text. Dark grey indicates a SE>0.4% and white a SE<0.2%, while the contour lines of the growth rate are represented in the same palette of the colour-maps in Section 4.The diagonal shades of grey and white are consistent with our narrative: the growth profiles of countries are shaped by the interplay of fitness and the different growth drivers.

## References

[1] Smith A., Strahan W., Cadell T. (1776). An Inquiry into the Nature and Causes of the Wealth of Nations.

[2] Helpman E. (2005). The Mystery of Economic Growth.

[3] Aghion P., Howitt P. (1998). Endogenous Growth Theory.

[4] Acemoglu D. (2009). Introduction to Modern Economic Growth.

[5] Barro R.J., Sala-i-Martin X. (2004). Economic Growth.

[6] Tacchella A., Cristelli M., Caldarelli G., Gabrielli A., Pietronero L. (2012). A new metrics for countries’ fitness and products’ complexity. Sci. Rep..

[7] Teece D.J., Pisano G., Shuen A. (1997). Dynamic capabilities and strategic management. Strat. Manag. J..

[8] Solow R.M. (1956). A contribution to the theory of economic growth. Q. J. Econ..

[9] Arrow K.J. (1962). The economic implications of learning by doing. Rev. Econ. Stud..

[10] Romer P.M. (1986). Increasing returns and long-run growth. J. Polit. Econ..

[11] Romer P.M. (1990). Endogenous technological change. J. Polit. Econ..

[12] Lucas R.E. (1988). On the mechanics of economic development. J. Monetary Econ..

[13] Aghion P., Howitt P. (1992). A model of growth through creative destruction. Econometrica.

[14] Frankel J.A., Romer D.H. (1999). Does trade cause growth?. Am. Econ. Rev..

[15] Bernard A.B., Eaton J., Jensen J.B., Kortum S. (2003). Plants and productivity in international trade. Am. Econ. Rev..

[16] Lucas R.E. (1993). Making a miracle. Econometrica.

[17] Acemoglu D., Robinson J. (2012). Why Nations Fail: The Origins of Power, Prosperity, and Poverty.

[18] Murphy K.M., Shleifer A., Vishny R.W. (1989). Industrialization and the big push. J. Polit. Econ..

[19] Krugman P. (1991). History versus expectations. Q. J. Econ..

[20] Matsuyama K. (1991). Increasing returns, industrialization, and indeterminacy of equilibrium. Q. J. Econ..

[21] Hirschman A.O. (1958). The Strategy of Economic Development.

[22] Penrose E. (1959). The Theory of the Growth of the Firm.

[23] Teece D.J., Dosi G., Winter S. (1994). Understanding corporate coherence. Theory and evidence. J. Econ. Behav. Organ..

[24] Abramovitz M. (1986). Catching up, forging ahead, and falling behind. J. Econ Hist..

[25] Fagerberg J., Srholec M. (2017). Capabilities, economic development, sustainability. Camb. J. Econ..

[26] Lall S. (1992). Technological capabilities and industrialization. World Dev..

[27] Kremer M. (1993). The O-ring theory of economic development. Q. J. Econ..

[28] Sutton J. (2002). Rich trades, scarce capabilities: Industrial development revisited. Econ. Soc. Rev..

[29] Sutton J. (2012). Competing in Capabilities: The Globalization Process.

[30] Sutton J., Trefler D. (2016). Capabilities, wealth, and trade. J. Polit. Econ..

[31] Hausmann R., Hwang J., Rodrik D. (2007). What you export matters. J. Econ. Growth.

[32] Dalmazzo A., Pekkarinen T., Scaramozzino P. (2007). O-ring Wage Inequality. Economica.

[33] Zaccaria A., Cristelli M., Tacchella A., Pietronero L. (2014). How the taxonomy of products drives the economic development of countries. PLoS ONE.

[34] Hidalgo C.A., Klinger B., Barabási A.L., Hausmann R. (2007). The product space conditions the development of nations. Science.

[35] Ferrarini B., Scaramozzino P. (2016). Production complexity, adaptability and economic growth. Struct. Chang. Econ. Dyn..

[36] Pugliese E., Chiarotti G.L., Zaccaria A., Pietronero L. (2017). Complex economies have a lateral escape from the poverty trap. PLoS ONE.

[37] Sbardella A., Pugliese E., Pietronero L. (2017). Economic development and wage inequality: A complex system analysis. PLoS ONE.

[38] Boltho A., Carlin W., Scaramozzino P. (2018). Why East Germany did not become a new Mezzogiorno. J. Comp. Econ..

[39] Zaccaria A., Cristelli M., Kupers R., Tacchella A., Pietronero L. (2016). A case study for a new metrics for economic complexity: The Netherlands. J. Econ. Interact. Coord..

[40] Zaccaria A., Mishra S., Cader M.Z., Pietronero L. (2018). Integrating Services in the Economic Fitness Approach.

[41] Cristelli M., Tacchella A., Pietronero L. (2015). The heterogeneous dynamics of economic complexity. PLoS ONE.

[42] Cristelli M., Tacchella A., Cader M., Roster K., Pietronero L. (2017). On the Predictability of Growth.

[43] Tacchella A., Mazzilli D., Pietronero L. (2018). A dynamical systems approach to gross domestic product forecasting. Nat. Phys..

[44] Tacchella A., Cristelli M., Caldarelli G., Gabrielli A., Pietronero L. (2013). Economic complexity: Conceptual grounding of a new metrics for global competitiveness. J. Econ. Dyn. Control.

[45] Cristelli M., Gabrielli A., Tacchella A., Caldarelli G., Pietronero L. (2013). Measuring the intangibles: A metrics for the economic complexity of countries and products. PLoS ONE.

[46] Hidalgo C.A., Hausmann R. (2009). The building blocks of economic complexity. Proc. Natl. Acad. Sci. USA.

[47] Balassa B. (1965). Trade Liberalisation and “Revealed” Comparative Advantage. Manch. Sch..

[48] Gaulier G., Zignago S. (2010). Baci: International Trade Database at the Product-Level (the 1994–2007 Version).

[49] Caldarelli G., Cristelli M., Gabrielli A., Pietronero L., Scala A., Tacchella A. (2012). A network analysis of countries’ export flows: Firm grounds for the building blocks of the economy. PLoS ONE.

[50] Pugliese E., Zaccaria A., Pietronero L. (2014). On the convergence of the Fitness-Complexity Algorithm. arXiv.

[51] Barro R.J. (1991). Economic growth in a cross section of countries. Q. J. Econ..

[52] Mankiw N.G., Romer D., Weil D.N. (1992). A contribution to the empirics of economic growth. Q. J. Econ..

[53] Nadaraya E.A. (1964). On estimating regression. Theory Probab. Appl..

[54] Feenstra R.C., Inklaar R., Timmer M.P. (2015). The Next Generation of the Penn World Table. Am. Econ. Rev..

[55] Group W.B. (2017). World Development Indicators 2017.

[56] Feenstra R.C., Lipsey R.E., Deng H., Ma A.C., Mo H. (2005). World Trade Flows: 1962–2000.

[57] Barro R.J., Sala-i-Martin X., Blanchard O.J., Hall R.E. (1991). Convergence Across States and Regions.

[58] Acemoglu D., Dell M. (2009). Beyond neoclassical growth: Technology, human capital, institutions and within-country differences. Am. Econ. J. Macroecon..

[59] Quah D.T. (1996). Twin peaks: Growth and convergence in models of distribution dynamics. Econ. J..

